# Hysteria, Catalepsy, Metastasis of Hearing, &c.

**Published:** 1842-11-26

**Authors:** 


*Hysteria, Catalepsy, Metastasis of Hearing, &c.*—A young unmarried girl, whose health had been long delicate, became subject to violent hysterical fits, in consequence, it seemed, of the irritation produced by a seton in the side, which had been inserted for the relief of obstinate pleuritic symptoms. The fits usually came an hour or two after eating. On one occasion she entirely lost her power of speech, so that she could not articulate a single word—this state lasted for three days, and then the speech returned after an hysterical seizure. Not long afterwards, she experienced her first attack of distinctly-formed catalepsy; and from this period the paroxysms of this disease, and of hysteria generally, recurred several times in the course of twenty-four hours, the one alternating with the other. While she was in the cataleptic state, there seemed to be a complete insensibility of the entire surface of the body, so that it might be pinched or pricked without causing pain;—the limbs and body retained whatever position they were placed in; the movements of breathing were almost quite imperceptible; the pulse at the wrist could scarcely be felt at all, while that at the heart was exceedingly feeble and indistinct. This state sometimes lasted for one, two or three hours at a time, the patient however not remaining all the time in this death-like repose; for every ten or fifteen minutes she would cough once or twice, and then a movement of some part of the body followed, or she would even suddenly start up, leap out of bed, climb upon a chair or table, &c. and there perhaps assume an attitude that was "ordinairement fatigante," but yet "toujours gracieuse:" her features on such occasions always wore an air of "douceur et satisfaction." In this state she would remain until a fit of coughing came on, or till she was removed to bed by her attendants. One curious circumstance in this, as in other cases of somnambulism, is, that although the eyes be quite closed, the person avoids with the greatest nicety any impediments in their way, and perform feats which they would not even attempt to do in their waking state. On one occasion she rushed to the window, opened it, and was on the point of throwing herself out, when she fell into a cataleptic paroxysm, with one foot out of the window, and the other resting on the ground: in this position she remained until she was replaced in bed.

The cessation of each cataleptic fit was always indicated by the return of the respiratory movements, which gradually became more and more decided, until the patient opened her eyes and recovered her intelligence.

During one of these attacks, Dr. Duvard observed, that when he applied his hand to the epigastrium, the countenance of the girl gave expression of decided suffering, and to his surprise he found that if he applied his mouth to this region,—or, as subsequently was the case, to the soles of the feet or to the palms of the hands,—and spoke to her, she answered questions with perfect correctness, although she appeared deaf to any auditory impressions on the ear itself. It was not even necessary that the mouth should be applied directly to the *impressionable* parts; the intervention of a stick or of a rod of iron, &c. the one end of which was applied to the epigastrium or to the palms or soles, and the other end to the lips of the speaker, did not at all prevent the patient from hearing what was addressed to her, as her rational answers to any questions put in this manner abundantly testified. Dr. D. confesses that he had often read of similar occurences as this in books, but that he never felt inclined to attach any credit to the reports of them till he met with the present case. . . . . .

"There was no sensibility in the integuments of any part of the body, except of the epigastrium and of the palms of the hands and the soles of the feet. The skin might be pricked or pinched, the hair might be pulled out by the roots, the nostrils might be irritated, &c. without the patient exhibiting any symptoms of pain or uneasiness. But if the epigastrium, or the palms or the soles were even tickled gently with a feather, she immediately drew back, and her features indicated that something offended her. When the ball of a charged Leyden jar was applied to any of the sensitive parts, she at once made a violent start, and sometimes awoke up in a moment; but, strange to say, she remained quite motionless and passive even when the jar was discharged upon any other part of the body. (!)

"The senses of taste, too, and smell were singularly perverted as well as that of hearing. The most pleasant or the most nauseous substances might be placed upon the tongue, and the nostrils might be stuffed with assafœtida or tobacco, or irritated with hartshorn or æther, without apparently any sensation being excited; but if any sapid or strongly odourous substance was placed upon or approached to any of the sensitive regions, the countenance of the patient at once indicated that she was aware of it, and, if questioned, she answered at once whether it was pleasant or not, and could even sometimes tell the name of the substance used. When snuff was put upon her hand, she at once began to sneeze, although it produced no effect when pushed up the nostrils." (!)

There was, however, no transport of vision, nor yet any *clair-voyance*, with Mademoiselle Melanie.

Dr. D. subsequently lost sight of his patient; but he heard that the hysterical and cataleptic attacks had become much less frequent and severe than formerly. All medical treatment seemed to have been quite unavailing.—*Med. Chir. Rev.*, Oct. 1842, *from Gaz. Med.*

*Remarks.*—Cases like the preceding are certainly very *droll*, to say the least of them; and it will certainly puzzle the most knowing savant of the present day to account for some of the phenomena which they exhibit. There is one way, indeed, of solving the difficulty, and that is to deny *in toto* all the alleged facts, and to call all such statements by the very comprehensive appellations of *humbug* and tomfoolery.

But be it remembered, that excessive incredulity may be quite as ridiculous as extravagant credulity. It is no good reason why we should utterly disbelieve any alleged fact, because we cannot explain its occurrence, or because certain circumstances or phenomena connected with it seem at variance with our ordinary experience. There are so many mysteries in the phenomena of life, which are, and will probably always be, utterly inexplicable, that it is no easy thing to say where our belief is to stop. Far be it from us to lend a complacent ear to the extravagancies which have been so absurdly exhibited of late years on the subject of animal magnetism: they are too ridiculous to last but as a " nine month's wonder ;" but this is no sufficient reason for flatly denying, or smartly ridiculing, the reports of all such cases as the preceding one.—*Rev.*

